# Population genomic insights into variation and evolution of *Xanthomonas oryzae* pv. *oryzae*

**DOI:** 10.1038/srep40694

**Published:** 2017-01-13

**Authors:** Samriti Midha, Kanika Bansal, Sanjeet Kumar, Anil Madhusoodana Girija, Deo Mishra, Kranthi Brahma, Gouri Sankar Laha, Raman Meenakshi Sundaram, Ramesh V. Sonti, Prabhu B. Patil

**Affiliations:** 1 CSIR-Institute of Microbial Technology, Chandigarh, 160036, India; 2 CSIR-Centre for Cellular and Molecular Biology, Hyderabad, 500007, India; 3 Bayer BioScience Pvt. Ltd., Hyderabad, 500081, India; 4 ICAR-Indian Institute of Rice Research, Rajendranagar, Hyderabad, 500030, India

## Abstract

*Xanthomonas oryzae* pv. *oryzae ( Xoo*) is a serious pathogen of rice causing bacterial leaf blight disease. Resistant varieties and breeding programs are being hampered by the emergence of highly virulent strains. Herein we report population based whole genome sequencing and analysis of 100 *Xoo* strains from India. Phylogenomic analysis revealed the clustering of *Xoo* strains from India along with other Asian strains, distinct from African and US *Xo* strains. The Indian *Xoo* population consists of a major clonal lineage and four minor but highly diverse lineages. Interestingly, the variant alleles, gene clusters and highly pathogenic strains are primarily restricted to minor lineages L-II to L-V and in particularly to lineage L-III. We could also find the association of an expanded CRISPR cassette and a highly variant LPS gene cluster with the dominant lineage. Molecular dating revealed that the major lineage, L-I is youngest and of recent origin compared to remaining minor lineages that seems to have originated much earlier in the past. Further, we were also able to identify core effector genes that may be helpful in efforts towards building durable resistance against this pathogen.

Rice is the staple food for more than half of the human population. The gram-negative bacterium, *Xanthomonas oryzae* pv. oryzae (*Xoo*) is a serious problem in rice cultivation. *Xoo* infections are not only endemic to Asia and West Africa, but also have been reported from Australia and Latin America^1^. The infection of the pathogen in the xylem tissues of rice leads to leaf blight symptoms, that were first characterized back in the late 19^th^ century^2^,^3^. Introduction of Resistance (R) genes into rice cultivars has been considered to be the best management option for *Xoo*, and in this direction, at least 40 different R genes of rice have been identified till date against *Xoo*^4^. But every R gene is not efficient against every race of *Xoo*, due to the co-evolution of the pathogen along with the host^5^.

India is the second largest producer and also a major centre of diversity of rice. There are reports of some of the strains that can breakdown most of the major R genes deployed for resistance to *Xoo* in India^5^,^6^,^7^,^8^,^9^,^10^,^11^. Hence, a comprehensive understanding of the genetic diversity of the population of *Xoo* from India and its relationship with strains from the rest of the world is necessary. However, earlier efforts in this direction have been primarily limited to non-sequence based hyper-variable markers and few housekeeping genes^12^. Apart from resolving the relationship, there is also a need to study evolution of gene(s) that are known to be important for virulence, pathogenicity and fitness.

Advent of genomics era has revolutionized the field of bacteriology. Now by genome sequencing, we can generate and access complete genotype of an organism at an unprecedented rate and scale. Genome sequences of *Xoo* strains from other part of Asia, Africa and USA are already available. Apart from type strain of species *X. oryzae*, no other strain from India has been sequenced. Herein, we carried out whole genome sequencing of 100 *Xoo* strains, collected from 19 rice cultivating states in India in the last two decades. The pathotype information for 46 of these strains is available and they have been classified into eleven pathotypes that were assigned based on their reaction towards ten major resistance genes of rice^5^.

Apart from understanding the relationship of the Indian strains to those present worldwide, the present study allowed us to gain insights into the origin of lineages, pathotypes and highly pathogenic strains from India. Further, we were also able to analyze the evolutionary history of genes known to be important for virulence and pathogenesis. The study also provided novel insights into the origin of the closely related pathovar *Xanthomonas oryzae* pv. *oryzicola ( Xoc*) that specifically infects parenchymatous tissue. This mega-genomic resource would be invaluable in surveillance of the pathogen and future comparative studies of this phytopathogen.

## Results

### Whole genome sequencing and phylogenomic status of *Xoo* strains

We sequenced the whole genomes of 100 *Xoo* strains and one *Xoc* strain BXOR1. The raw reads for all strains were de novo assembled into genomes with <500 contigs and >100x coverage. The *Xoo* strains have an N50 value of ~18–24 kb while BXOR1 assembly has N50 value 46.7 kb. All the sequenced strains show conservation in genome size and number of genes. Assembly statistics and annotation features of these genomes are listed in Supplementary Table S1.

**Figure 1:**
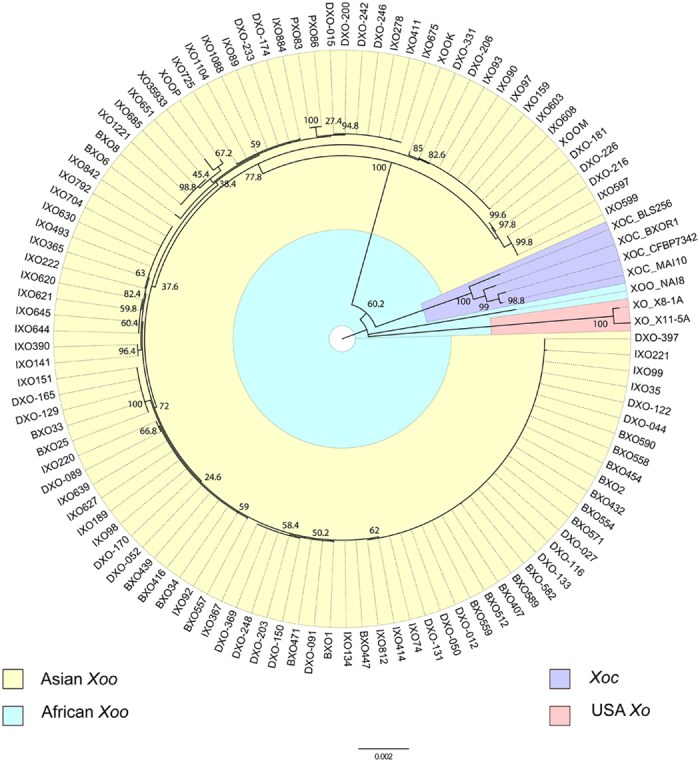
Phylogenomic markers based tree of *Xanthomonas oryzae* strains. 31 phylogenomic marker genes were extracted from the 113 genomes, concatenated and aligned using ClustalW algorithm. Maximum likelihood tree of conserved phylogenomic marker genes was constructed using General Time Reversible model (Gamma distributed with Invariant sites (G + I)). Bootstrap values shown on the nodes are percentage of 500 replicates. The scale bar (0.002) indicates the number of nucleotide substitutions per site. Clades from different geographical locations are coloured differently.

Sequenced *Xoo* strains have Average Nucleotide Identity (ANI) values >99% with the *Xanthomonas oryzae* type strain 35933 (XO35933) which are above the cut-off of 96% for delineation of novel species^13^. We constructed a phylogenomic marker genes based tree of sequenced strains along with their relatives whose sequences are available publically^14^. It suggested that the 106 *Xoo* strains that include 100 Indian *Xoo* strains from the present study, type strain belonging to India and five strains from other parts of Asia from the public domain form a distinct cluster that is closer to pathovar *Xoc* than African *Xoo* and USA *Xo* strains. Interestingly, *Xoc* strains appear to be a variant lineage of *Xoo* population (Fig. 1).

### Clonal analysis reveals a major clonal lineage and minor diverse lineages

**Figure 2:**
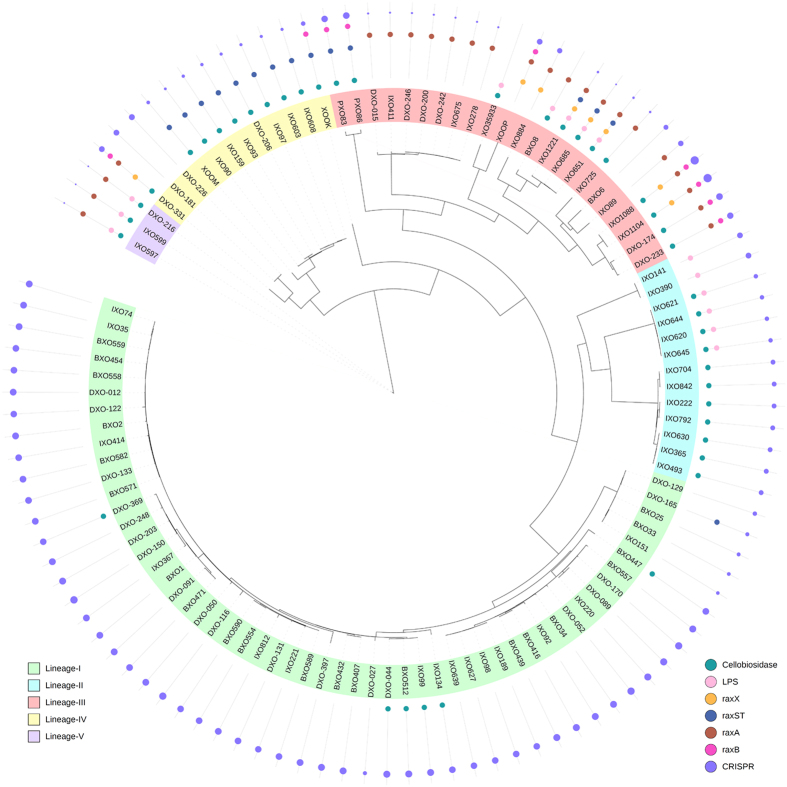
ClonalFrameML tree obtained from genomic sequences of 100 Indian *Xoo* strains and six *Xoo* strains from other parts of Asia. Genomes of 106 strains were aligned and core genome was analysed using ClonalFrameML to obtain a tree considering recombination. Different lineages inferred are coloured differently. On the tree in circular way, information on seven different genes/clusters is marked. Moving outward in the circles, the strains are marked with having BXO8 type cellobiosidase allele (1), BXO8 type LPS cassette (2), genes having non-synonymous changes in *raxX* (3), *raxST* (4), *raxA* (5), *raxB* (6) and diameter of outermost circle indicating the variation in the number of CRISPR spacers.

As the 106 *Xoo* strains formed a clade, distinct from African, USA and *Xoc* strains, this major group may be freely recombining and exchanging genes. Hence, we carried out an in-depth phylogenetic analysis specifically using regions not affected by recombination (see methods). Whole genome based tree (Fig. 2) showed the presence of five distinct lineages with lineage L-I encompassing >50% (55/100) of strains, a predominant lineage with high clonality. The rest of the four lineages, constitute the other half of *Xoo* population and are highly diverse than lineage L-I.

**Figure 3:**
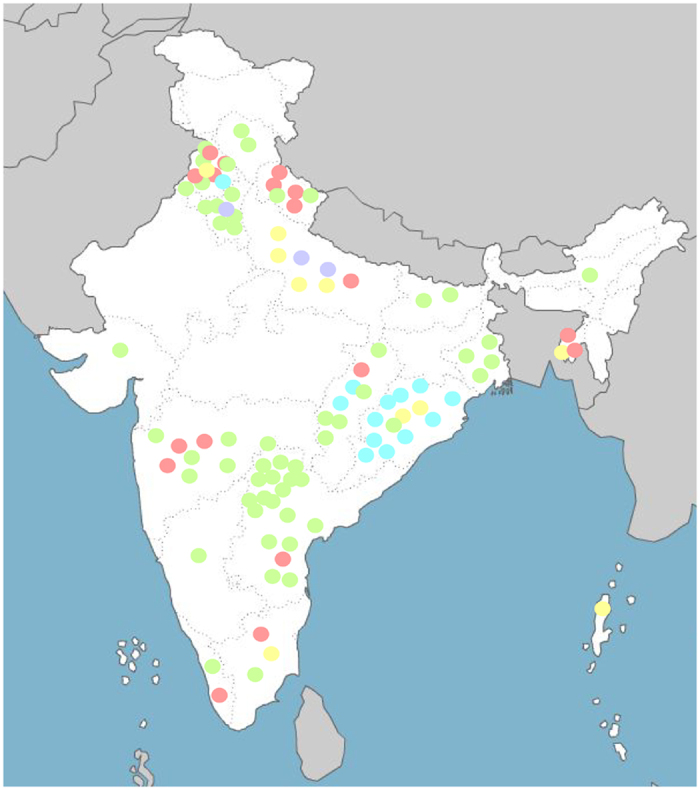
Map of India indicating the geographical origin of *Xanthomonas oryzae* pv. *oryzae* strains under study. Strains belonging to different lineages are colored differently; Lineages L-I (in green), L–II (in blue), L-III (in pink), L-IV (in yellow) and L-V (in purple) (Map has been adapted from http://d-maps.com/carte.php?num_car=4182&lang=en).

It is interesting to note that lineages L-I and L-II are exclusively constituted by Indian *Xoo* strains. Philippine strains *Xoo* PXO86 (PXO86), *Xoo* PXO83 (PXO83) and *Xoo* PXO99A (XOOP) belong to lineage L-III. Japanese *Xoo* strain, *Xoo* MAFF 311018 (XOOM) belongs to lineage L-IV, while Korean strain *Xoo* KACC 10331 (XOOK) is an out-group to the clade consisting of lineages L-I to L-III. We also looked at the geographical distribution of various lineages in India (Fig. 3). While southern and eastern regions of the country are mainly dominated by lineages L-I, L-II and L-III, all five lineages were found in the northern region. The lineage L-II is restricted to the eastern parts of the country, excepting for one isolate from the northern region.

### Role of recombination in shaping the diversity of *Xoo* lineages

Since the ClonalFrameML tree is based on clonal regions of the genome, the tree and branch length relationship and diversity we observe, is based on mutation rate alone. The analysis showed that the ratio of recombination to mutation (R/θ) is 0.2586, the average length of the recombined fragment (δ) is 576 bp and average distance between donor and recipient (ν) is 0.015. Thus, the mutations are ~3.86 times more frequent than recombination, while the impact of recombination over mutation is 2.2 times higher towards the evolution of these strains.

**Figure 4:**
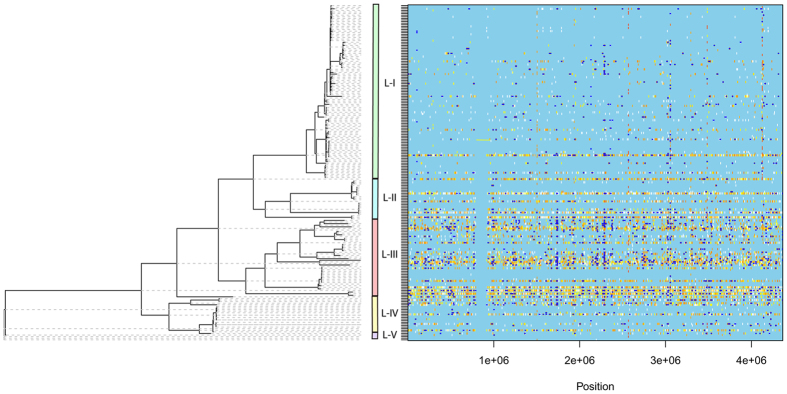
Graphical representation of recombinational events in the *Xanthomonas oryzae* pv. *oryzae* strains. The image shows the phylogenomic relationship of the strains along with sites of recombination and substitutions. Each clade is compared to its most recent common ancestor for variations and represented in different colored bars. Recombination events are marked by dark blue horizontal bars and substitutions by vertical lines. Light blue vertical sites refer to no substitution, white sites to the non-homoplasic substitutions whereas any other colour refers to homoplasic substitutions, with increase in redness from white to red marks increase in homoplasy.

Graphical representation of recombinational events in these *Xoo* strains is shown in Fig. 4. The graph shows a higher density of variation in lineages L-II to L-V as compared to lineage L-I, which has a comparatively lesser number of recombinational events as well as substitutions. Lineage L-II shows a higher density of yellow-red vertical bars which is consistent with its (R/θ) value (0.1734), implying ~5.76 times more mutational occurrences as compared to recombinational events. For lineage L-III recombinational events are higher as well as the substitutions, with a final impact of recombination 2.4 times higher than mutations. Thus, the strains in lineage L-I are less diverse than the strains found in all other lineages. Interestingly, three highly diverse strains DXO-216, IXO599 and IXO597 that are from lineage L-V are more basal and probably constitute the ancestral lineage.

### Clonal analysis reveals ancestral and lineage associated pathotypes

For 46 strains that are part of this study, detailed pathotype information is available and they belong to eleven pathotypes^5^. The distribution of these strains in the phylogenetic tree was assessed. Two strains (IXO1088 and IXO1104) belonging to the most virulent pathotype XI, which can break down all 10 resistance genes, are restricted to highly diverse lineage L-III. Similarly, strains belonging to pathotype III-V are restricted to lineage L-I, while strains belonging to pathotype VI, II and IX are restricted to lineages L-II, L-III and L-IV respectively. Four pathotypes (I, VII, VIII and X) do not show any association or restriction to a particular lineage. Hence, these four may be ancestral, while others may be of recent origin.

Interestingly, the five lineages also differed in their reaction towards two major resistant genes, *xa5* and *xa13*. These are recessive resistant genes, where the recessive allele has mutations in the coding region (*xa5*) or promoter region (*xa13*) that makes them recalcitrant for promoting bacterial growth and proliferation^15^,^16^. Looking at the phylogeny of *Xoo* strains pathotyped earlier^5^, it showed that strains compatible with *xa5* are grouped in lineages L-I and L-III, while the strains compatible with *xa13* are clustered in lineages L-II, L-IV and L-V.

### Molecular dating of *Xoo* lineages

In order to estimate the age of establishment of *Xoo* lineages in India, we deployed Bayesian method approach. We first tested for the presence of a sufficient temporal signal in the dataset using root-to-tip regression approach implemented in TempEst^17^. Then we checked for both the strict and relaxed clock model to know which fits better for our data (Supplementary Table S2). The relaxed clock model analysis results showed higher likelihood log values with both harmonic mean and stepping stone methods for our data. Finding a better performance for relaxed clock model, we used this model to estimate the age of various nodes to determine the emergence time for *Xoo* strains (Supplementary Fig. S1). We used a mutation rate estimated in *Xanthomonas* pathogens earlier (2 × 10^−5^ mutations per gene per year)^18^ to define the priors in the analysis. The analysis suggested the emergence of these strains from a common ancestor around 0.97 [95% HPD (highest-probability density): 0.922–1.00]) Myr ago and emergence of lineage L-I around 0.3 [95% HPD: 0.267–0.354] Myr ago.

### Variation across candidate virulence genes and hypervariable loci

Plant cells recognize various microbial signature molecules such as flagellin, lipopolysaccharide, etc. that act as Microbial/Pathogen Associated Molecular Patterns (MAMP/PAMP)^19^. To circumvent this recognition and hence triggered immunity, bacteria release specialized protein molecules directly into the host cell known as type III effectors using its well-evolved type III secretion system^20^. In turn, plants have also evolved specialized Resistance (R) genes that act in response to effector molecules^21^. Herein, we analyzed variation in well-known genes/cassettes that are associated with either damage associated molecular pattern (DAMP) (e.g. cellobiosidase gene) or PAMP (e.g. *raxX, fliC*, lipopolysaccharide cassette) and type III effectome that is known to counteract PAMP triggered immunity (PTI).

### Cellobiosidase

**Figure 5:**
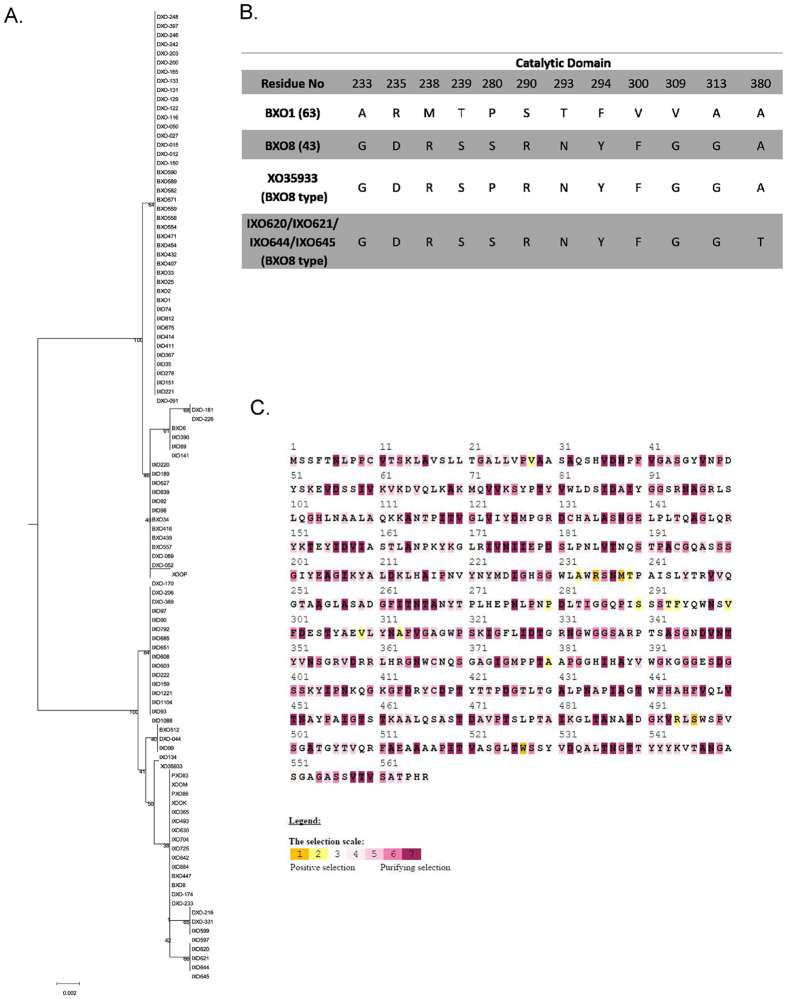
Phylogenetic analysis of cellobiosidase encoding gene. Cellobiosidase protein sequences of 100 Indian *Xoo* strains and six *Xoo* strains from other parts of Asia were aligned and phylogenomic tree was constructed using Neighbour Joining method. Bootstrap values shown on the nodes are percentage of 500 replicates. The scale bar (0.002) indicates the number of amino acid substitutions per site (**A**), variation in the amino acid residues of catalytic domain are listed in tabular form (**B**) and selection pressure for each residue position is depicted with colour variations where an increase in yellow colour represents increase in the positive selection pressure on the residue (**C**).

Cellobiosidase, secreted by type II secretion system, is a major pathogenicity determinant of *Xoo*^22^. The phylogenetic tree of the *cbsA* gene of 100 Indian *Xoo* strains and six other Asian strains is not in congruence with the genome based phylogeny (Fig. 5A). There are two different alleles of *cbsA* (referred as BXO1 type and BXO8 type), marked on Fig. 2. Further looking at the amino acid level in the catalytic domain (33–457 aa), it showed the changes in 12 amino acid residue positions in both alleles (Fig. 5B). We further checked for the dN/dS ratio, which is 0.667, comparatively much higher than for a housekeeping gene *rpoB*, for which it is 0.001. We also checked for the selection pressure on amino acid residues using Selecton server^23^. Figure 5C shows the different amino acid residues and their positions, which are under high selection pressure.

### *fliC* gene

The *fliC* gene encodes for flagellin which serves as a PAMP^24^. We also looked for the variation at this locus in the *Xoo* strains and analysis revealed that *fliC* gene is highly conserved amongst the strains. The protein sequences encoded by this gene in 106 *Xoo* strains are highly identical except for changes at two amino acid residue in XOOK. We also found the presence of two copies of *fliC* gene in XOOP, encoding for identical copies of protein.

### *raxX* and *raxSTAB*

The gene *raxX* is a recently discovered PAMP in *Xoo* that encodes for a peptide which is recognized by resistance gene *Xa21*^25^. Mutation in *raxX* restores the ability to cause disease in *Xa21* containing host plants. To explore the *raxX* sequences in Indian *Xoo* strains, we constructed a RaxX protein tree (Supplementary Fig. S2). The tree revealed that three of the *Xoo* strains (IXO651, IXO685, and IXO1221) have highly variant *raxX* allele closer to *Xoc* strains, rather than other *Xoo* strains. These three strains are already reported to break down the *Xa21* mediated resistance in the rice^5^,^25^. These strains are also grouped together in the genome based tree and differ by ~20–40 SNPs. Interestingly the amino acid positions P44 and P48 which are known to be important for *Xa21* mediated immunity^25^, are variant in these strains in reference to BXO1. These non-conservative variations, where a hydrophobic amino acid (proline) is replaced by a hydrophilic amino acid (serine or threonine), are also similar to *Xoc* strains (Table 1).

As *raxST, raxA* and *raxB* are present in the genome as a single operon, adjacent to *raxX* gene, protein trees for RaxST, RaxA and RaxB were obtained and strains having variations in the four proteins in reference to BXO1 allele are marked in Fig. 2. Similar to the *raxX* sequences, the *raxST* gene in IXO651, IXO685and IXO1221 is also having high similarity to *Xoc* than other *Xoo* strains. Interestingly except for one strain DXO-165 in lineage L-I, all the non-synonymous changes in *rax* genes have taken place on diverse lineages L-II to L-V. We also looked into variation in five other genes (*raxC, raxH raxP, raxQ* and *raxR*) related to this cluster. The variations are marked in the Supplementary Fig. S3, which are mostly clustered in lineages L-III to L-V, except for *raxQ* which showed variation in lineage L-II.

### Lipopolysaccharide

Lipopolysaccharide (LPS) is a constituent of the outer membrane of gram-negative bacteria and LPS gene clusters are hypervariable because of horizontal gene transfer. Published studies in *Xoo* have reported two different LPS cassette types; BXO1 type and BXO8 type^26^. Interestingly all the strains of lineage L-I have BXO1 type LPS cassette, while 13 strains sequenced in this study (BXO8, IXO1221, IXO651, IXO685, IXO597, IXO599, DXO-216, IXO390, IXO141, IXO621, IXO644, IXO645 and IXO620) and XO35933 that belong to diverse lineages, showed the presence of BXO8 type LPS cassette (Fig. 2).

### Type III Effectors

**Figure 6:**
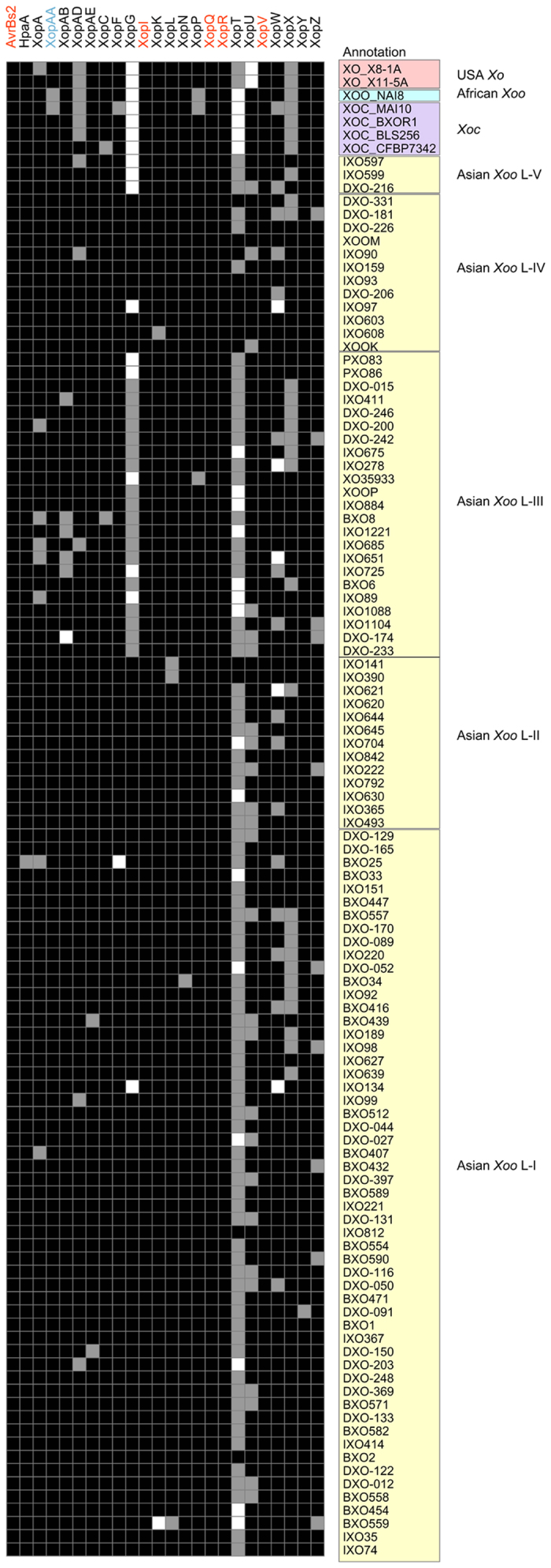
Heatmap depicting the conservation of 24 type III effectors in *Xanthomonas oryzae* strains. Presence of an effector is marked with black colour, absence in white and effectors with partial sequences/contig break/disruption or frameshift mutation are marked in grey. In the upper row with the name of type III effector, the effectors that are conserved in all strains are marked with red font and an effector conserved only in 106 Asian strains (100 Indian *Xoo* strains sequenced and six *Xoo* strains from other parts of Asia) is marked with cyan colour. Strain names on the right side are arranged according the phylogenomics relationship.

Type III Effectors (T3Es) of *Xanthomonas* play an indispensable role in disease development. Owing to the repetitive nature of TAL (transcription activator-like) effectors, it is difficult to study them in draft genomes. Hence we focused only on the non-TAL effectors of *Xoo*. We checked for the presence and conservation of T3E repertoire in *Xoo* strains by analyzing the 24 non-TAL T3Es, which are listed in www.xanthomonas.org to be associated with *Xoo*. Out of the 24 non-TAL T3Es, only five effectors (AvrBs2, XopI, XopQ, XopR and XopV) make the core effectome of all 113 *Xo* strains (including Indian and few Asian, African and USA strains) of *Xanthomonas oryzae* and are conserved throughout, while XopAA is also conserved in all Asian *Xoo* strains studied here. A detailed list of presence/absence and changes in the T3Es in all 113 strains is provided in Supplementary Table S3. Figure 6 shows a heatmap for T3E conservation in the 113 *Xo* strains.

### Lineage associated variation in number of repeats at a CRISPR locus

Clustered Regularly Interspaced Short Palindromic Repeats (CRISPR) represent the acquired immunity by bacteria, where acquired spacer regions from phages or plasmid sequences act as the inheritable memory and help in recognition of cognate protospacers in the invasive elements limiting their attack^27^,^28^. Hence an evolved CRISPR cassette can provide immunity from phages that use LPS as a receptor and also restrict the rate of horizontal gene transfer through extra-chromosomal elements like plasmids. Hence it is pertinent to study variation in CRISPR in *Xoo* population. Two strains, BXO8 and XO35933 did not show any spacer regions, but the entire locus containing *cas3, cas5, cas8c, cas7, cas4, cas1* and *cas2* is present in both draft genomes. Remaining *Xoo* strains showed the presence of CRISPR locus with a highly variable number of repeats and spacer regions (Fig. 2) that belong to CRISPR type I. The CRISPR sequences and number of spacers are listed in Supplementary Table S4.

The direct repeats are 31 bp in length and number of spacer sequences varies from 0–108. There is an association of higher and uniform number of repeats with members of the major lineage L-I, while members of minor lineages have less or highly variable number of repeats. Highly virulent pathotype XI strains IXO1088 and IXO1104 showed the presence of maximum number of spacers (108). CRISPR sequences for both the strains are highly similar. We also compared the alignment of repeats of CRISPR sequences of IXO1088 with Philippine race (XOOP), Korean race (XOOK), Japanese race (XOOM) and Supplementary Fig. 4 shows the alignment of spacer sequences in the four strains.

### Plasmid detection and analysis

We also checked for the presence of plasmid in the raw reads and 11/100 strains showed the presence of plasmid sequences in the data. Interestingly, seven of the strains are from major lineage L-I, while remaining four strains are from minor lineages. With BLAST analysis of plasmids assembled using plasmidSpades^29^, we could find four different type of plasmids in these strains; *Xanthomonas albilineans* str. GPE PC73, plasmid plasmIII^30^ (BXO1, IXO35, IXO704, IXO842), *Xanthomonas campestris* pv. campestris B1459, plasmid I^31^ (IXO35, IXO74, IXO97, DXO-050, DXO-091, DXO-133, DXO-206), *Xanthomonas citri* subsp. citri strain 306, plasmid pXAC64^32^ (IXO134), *Burkholderia vietnamiensis* G4, plasmid pBVIE04^33^ (DXO-206). A compiled list of the length, assembly statistics of these plasmids and their BLAST result is provided in Supplementary Table S5. Further BLAST analysis of the plasmids identified in the 100 genomes in genomic sequences showed the presence of *Xanthomonas albilineans* str. GPE PC73, plasmid plasmIII in BXO447 and *Burkholderia vietnamiensis*, G4 plasmid pBVIE04 in BXO1 strain also.

## Discussion

Being a staple food of half of the world’s population, rice improvement and protection is of paramount importance. Further, the history of domestication of rice parallels with the birth of human civilization and advancement. Hence detailed understanding of phylogeny and evolution of its pathogens is important. Even though, India is a major region of diversity of rice and *Xoo*, whole genome based studies are markedly lacking. The present population genomics study clearly revealed that Indian *Xoo* strains along with few Asian strains form a lineage that is distinct from USA and African strains. Further, the Indian *Xoo* population exhibits epidemiological structure with a predominant clonal lineage and few minor diverse lineages. The analysis also suggested that *Xoc* may be a variant lineage that emerged from the *Xoo* population.

New studies using genomic data reveal that rate of evolution can be highly variable in pathogenic bacteria^34^. Hence it is necessary to test both strict and relaxed clock model with methods that allow incorporations of uncertainties in the inferences. This is particularly relevant for a pathogen of a staple crop like rice that is cultivated in millions of hectares and with multiple cropping seasons within a year in tropical regions. Accordingly our testing revealed that a relaxed clock model is more suitable in *Xoo* and there have been two waves of selection. One population consisting of strains belonging to a major clonal lineage is younger and of recent origin while the other population consisting of minor recombining lineages is diverging from a much longer time.

Recombination analysis on the *Xoo* population has identified a clonal lineage L-I, which is predominant in India and lineage L-III, which is highly diverse and consists of strains of pathotypes I, II and XI. Grouping of these three pathotypes together marks this lineage as the most virulent lineage among all that were identified in this study, as very few resistance genes are effective against these pathotypes^5^. Interestingly, pathotype X consists of strains that are incompatible with all the R genes used for pathotyping^5^. They were considered to have lost their virulence. As these strains are present in multiple lineages, it appears that loss of virulence has occurred multiple times. Interestingly the Indian *Xoo* strains show compatibility to rice genotypes containing either *xa5* or *xa13*, but not to both (except IXO1088 and IXO1104 belonging to pathotype XI)^5^. This implies that the switching of capability to break *xa5* from *xa13* has occurred twice in *Xoo* population, once in the ancestor of lineage L-III and once in the ancestor of lineage L-I.

Studying the phylogeny of determinants of pathogenicity, virulence and fitness is as important as understanding the strains phylogeny. Population genomic studies in particular provide opportunity to study variation in genes that encode proteins that act as PAMP/DAMP and also hypervariable genomic regions. One of the well-known PAMP in phytopathogen is flagellin which is under purifying selection in *Xoo*. Earlier studies have shown that a mutation in the gene coding for the cell wall degrading enzyme cellobiosidase leads to high virulence deficiency in *Xoo*^22^. Purified cellobiosidase is shown to induce innate immune responses and programmed cell death in rice tissues^22^. Our study revealed the presence of two alleles of *cbsA* (BXO1/BXO8 type) and one allele (BXO1 type) is replacing the other (BXO8 type) over time. Interestingly, the gene encoding for *cbsA* has acquired a frameshift mutation in *Xoc* and hence was reported to be missing in *Xoc*^35^. This suggests the strong selection pressure on *cbsA* gene in *Xoo* strains and pathovars.

In *Xoo*, several genes have been recognized that are required for *Xa21* activity of rice, which is the most effective R gene against the Indian population of *Xoo*^5^. They are known as *rax* (required for AvrXa21 activity), and a gene known as *raxX* encodes for a 60 aa peptide, which is tyrosine-sulfated by *raxST* and recognized by *Xa21* in rice for its activity^25^. Interestingly the limited SNPs amongst three strains IXO651, IXO685 and IXO1221 that have a variant *raxX* allele enabling them to evade the *Xa21* immunity, suggests their clonal nature and their recent origin. In this context, it is important to note that *Xa21* has been formally deployed only in the last 10–15 years. The IXO651 and IXO685 strains were isolated in 2006 from widely separated locations in India, and IXO1221 was isolated in 2011. It is unclear whether these strains have evolved in response to the recent deployment of this gene or whether they had arisen in response to some other selection pressure. Irrespective of the nature of selection, the *raxX* polymorphisms can be used to develop PCR based assays for detecting *Xoo* strains that are highly virulent on *Xa21* containing cultivars.

Similar to the *Xa21* mediated activity, flagellin sensing activity is also conserved in the rice plants, but as shown earlier the flagellin molecule produced in rice infecting *Xanthomonas* strains has a variant structure to avoid this detection^36^. While another *Xanthomonas* pathogen *X. campestris* has shown high level of polymorphism at this locus^37^, we could not find much differences in the rice pathogens. A very high level of similarity at this locus in all *Xoo* strains is suggestive of the high conservation of this newly gained trait in the population.

Lipopolysaccharide is well known to act as PAMP, virulence determinant and an elicitor of defense responses in plants^38^,^39^,^40^,^41^,^42^,^43^. *Xanthomonas* LPS locus is known to be hypervariable in nature^26^ and presence of two cassette types in *Xoo* population further supports this fact. BXO1 type LPS cassette is present in the majority of the population, while BXO8 type is present not only in the ancestral *Xoo* lineage and hyper virulent strains IXO651, IXO685 and IXO1221, but also in the old strain *X. oryzae* type strain XO35933. One remarkable finding is that the non-canonical BXO8 cassette is strikingly absent in lineage L-I. Besides orthologues of BXO8 type LPS cassette has also been identified in type strains of *X. axonopodis, X. citri* and *Xoc* strains^26^. Presence of a same type of LPS cassette in multiple pathovars and species, while restriction of the BXO1 type LPS cassette to *Xoo* strains only raises the possibility that the BXO8 type of cassette is the ancestral cassette for *X. oryzae*, while the BXO1 type cassette must have been acquired during later stages of *Xoo* evolution. LPS does not only act as PAMP, but is also known to be a receptor of bacteriophages^44^ and hence the variation can also be due to selection pressure from the phages.

T3Es are injected by pathogenic bacteria into the host cells to take control of host machinery. T3Es are known to be important to counteract PAMP induced defense responses by the host^45^. Thus the core effectome of the population can be an important resource for molecular and traditional breeding strategies to tackle the disease. In our study on *Xoo*, we have been able to identify T3Es (AvrBs2, XopI, XopQ, XopR and XopV) that are core to the whole *Xoo* population studied. Out of the five genes we identified as core, three were also listed as core T3Es in an earlier study based on PCR and dot-blot hybridization^12^. Interestingly, out of the five effectors common to all rice pathogens, 4 are also shared with beans pathogens^46^, 3 with tomato/pepper pathogens^47^,^48^ and one XopV is also shared by cassava pathogens^49^ and none with *X. campestris* pathovars^50^.

Various T3Es of *Xanthomonas* has been shown to interact in a distinct way inside the host plant. While some of them are involved in suppressing the innate immunity pathways of the plant^51^, others play a role in suppressing the defense mechanisms activated inside the host due to damage occurred by various cell wall degrading enzymes of bacteria^52^. Some T3Es also have target receptors inside the host that play intricate role in interfering with various signaling pathways^53^. Amongst the five T3Es identified to be conserved in this study, AvrBs2 is known to be required for the full virulence of bacteria^54^, XopQ is involved in suppressing the damage associated rice immune responses^52^, XopR plays a role in inhibiting the basal defense responses^55^, while detailed function of XopI and XopV still needs to be elucidated. Future similar population genomic based comparison of effectome of all *Xanthomonas* pathogens will be an intriguing area of research.

While CRISPR provides immunity to bacteria from phages, at the same time it will limit acquisition of novel genes through bacteriophages and plasmids. This has major implication on the genome dynamics and virulence of a pathogenic bacterium like *Xoo*. Hence it is important to check the presence and distribution of plasmid sequence in *Xoo* lineages. Interestingly, Asian *Xoc* strains and all African and USA *Xo* strains lack CRISPR loci as well as the CRISPR-associated genes^56^. Comparison of CRISPR cassette of Indian, Philippine, Korean and Japanese race showed that Indian race is closer to Philippine race as compared to Korean and Japanese.

It is striking to note that only in the major L-I lineage, most of the strains harbor a CRISPR cassette with >80 spacers. Interestingly, most of the strains encoding plasmid(s) are from lineage L-I. This suggests a highly evolved CRISPR locus, that might be central to providing immunity against invading phages and plasmids during the population expansion of lineage L-I. Also, only in major lineage L-I, all the strains have a variant BXO1 type of LPS locus, specific to *X. oryzae*, whose products again might have a role in protection against phages. Association of a large CRISPR cassette and a variant LPS gene cluster gives us a hint on the success of lineage L-I, and also suggests a promising way of controlling lineage L-I using bacteriophages. Such an association of CRISPR cassette with a predominant lineage and hypervirulent strains is intriguing.

Being a pathogen of a major staple crop seems to be a highly intricate evolutionary and ecological process. The predominant population of *Xoo* is clonal while the hyper variable counterpart forms a minor population. The latter also harbours highly virulent strains and highly variant allele(s)/gene cluster(s). In any case, considering the potential damage the minor lineages and variant strains can inflict, there is a need to track their movement and variation, along with effective deployment of resistance genes. This genomic resource will be invaluable in surveillance of *Xoo* by designing strain specific primers for quick PCR based diagnostic tools. However, considering the genome dynamics in *Xoo*, it is necessary to test such primers on much larger and new collection of strains. Specific mutations in the *raxX* gene are clearly associated with an ability to cause disease on *Xa21* containing cultivars. This information can be used to develop PCR based diagnostic tests for detecting such isolates that are highly virulent on *Xa21* containing rice lines.

## Material and Methods

### Genome sequencing

We have used two different sets of strains for this study, 46 strains from the Indian *Xoo* pathotype diversity study (2004–2009), where 11 different pathotypes were assigned to *Xoo* based on their reaction towards ten major resistance genes of rice^5^, and 54 strains from another old collection (1991–2014). We have included 2–5 representatives of each pathotype in the present study. A total of 100 *Xoo* strains, collected from diverse geographical locations of the country and a strain of *Xanthomonas oryzicola* pv. *oryzicola*, BXOR1 (listed in Supplementary Table S1), were grown on peptone sucrose agar (PSA) and genomic DNA was isolated using ZR Fungal/bacterial DNA isolation kit (Zymo Research Corporation, Orange, CA, USA). DNA quality was checked by running DNA samples on the 0.8% agarose gel and quantitation was done using Qubit 2.0 fluorometer (Invitrogen, Carlsbad, CA, USA). For sequencing on Illumina platform, the library was prepared using Nextera XT sample preparation kit (Illumina, Inc., San Diego, CA, USA) with dual indexing. Sample libraries were either normalized with the beads provided in the kit or quantitated by KAPA library quantification kit (KAPA Biosystems) using real time PCR and then loaded onto in-house Illumina MiSeq platform (Illumina, Inc., San Diego, CA, USA). The strains were sequenced using Illumina paired end sequencing technology (2 × 250).

### Assembly and Annotation

Assembly of the raw sequences (>100x coverage) was performed using CLC Genomics workbench 6.5 (CLC bio, Aarhus, Denmark) into contigs (<500). Annotation was done using PGAAP pipeline of NCBI. CRISPR sequences were recognized using CRISPR recognition tool^57^ and CRISPRFinder webserver^58^. Spacer sequences were compared using blastn. LPS cassette of BXO1 and BXO8 were retrieved from NCBI to find their homologs in *Xoo* strains. Type III effector (AvrBs2, XopC, XopF, XopG, XopI, XopK, XopL, XopN, XopP, XopQ, XopR, XopT, XopU, XopV, XopW, XopX, XopY, XopZ, XopAA, XopAB, XopAD, XopAE, XopA and HpaA) sequences of XOOM were retrieved from the list provided on www.xanthomonas.org and further used to find the homologs in *Xoo* strains.

### Phylogenetic analysis

Average Nucleotide Identity was calculated using JSpecies v1.2.1^13^. Phylogenomic tree of Indian *Xoo* strains along with other Asian strains [XOOK^59^, XOOM^60^, XOOP^61^, PXO86^62^, PXO83^63^, XO35933 (NCBI accession number: AXVI00000000), XOC_BLS256^64^], African strains (XOO_NAI8^65^, XOC_MAI10^65^, XOC_CFBP7342^62^) and the USA strains (X8-1A^56^, X11-5A^56^) was obtained using 31 phylogenomic marker genes^14^, extracted from each genome and concatenated. Mega 7.0 was used for obtaining multiple sequence alignment of the concatenated sequences as well as to obtain phylogenetic tree^66^. Maximum likelihood tree was constructed using General Time Reversible model (Gamma distributed with Invariant sites (G + I)) method with 500 bootstrap replications.

ClonalFrameML tool (which uses maximum likelihood inference) was deployed for identifying the recombined fragments and applying a correction for recombination in the final phylogeny inferred^67^. For obtaining tree with correction for recombination, genomes of 106 *Xoo* strains were aligned using MAUVE and a maximum likelihood tree was obtained using PhyML. MAUVE alignment^68^ and PhyML tree^69^ were further used to generate the ClonalFrameML tree and recombination parameters with 100 bootstrap replications (emsim = 100).

For phylogenomic inferences of different genes, protein sequences were aligned for RaxX, RaxST, RaxA, RaxB, RaxC, RaxH, RaxP, RaxQ, RaxR and cellobiosidase using Mega v6.0^70^. A neighbour joining tree was obtained for different protein alignment with 500 bootstrap replications using Mega v6.0.

### Molecular clock analysis

TempEst v1.5 was used to check for the temporal signal^17^. A maximum likelihood tree was associated with isolation dates of the strains using root-to-tip regression approach. A best fitting root option was used and correlation coefficient function was determined to be 0.372. For Bayesian analysis, *Xoo* genomes of 100 Indian *Xoo* strains and six *Xoo* strains from other parts of Asia were aligned using Mauve and the alignment was analysed using Mr. Bayes v3.2^71^. Testing of strict clock model and relaxed clock model [independent gamma rates (igr)] was done using harmonic mean method (ngen = 100000) and stepping stone method (ngen = 255000) and values for harmonic mean and maximum likelihood were compared. For dating analysis, Mr. Bayes was run with GTR substitution model with gamma-distributed rate variation across sites and a proportion of invariable sites for 1 M iterations (ngen = 1000000) and two parallel runs (nrun = 2). Molecular dating was done with clock rate (0.02, 0.004) per million year considering the mutation rate of 2 × 10^−5^ mutations per gene per year as earlier reported in *Xanthomonas*^18^ and assuming the average gene size as 1 kb. After confirming for convergence of two runs, parameters were summarized and results were obtained.

### Plasmid Detection

Presence of plasmids was predicted using Spades v3.8.0 with argument – plasmid based on the reads coverage^29^. The contigs (size >5 kb) predicted as plasmid by plasmidSpades were further tested using blastn in complete plasmid database as well as in nr database. Plasmids identified in any genome were further also manually checked for their presence in other 100 genomes sequenced.

## Additional Information

**Accession codes:** Genomic sequences have been submitted to NCBI GenBank. Genomic sequences of *Xanthomonas oryzae* pv. *oryzae* strains are available under accession number JXDM-JXHH and genomic sequences of *Xanthomonas oryzae* pv. *oryzicola* strain is available under accession number JXHI. Genome files with annotation of 101 strains are available at https://figshare.com/s/10e290cfe8a5f31858d3.

**How to cite this article**: Midha, S. *et al*. Population genomic insights into variation and evolution of *Xanthomonas oryzae* pv. *oryzae. Sci. Rep.* **7**, 40694; doi: 10.1038/srep40694 (2017).

**Publisher's note:** Springer Nature remains neutral with regard to jurisdictional claims in published maps and institutional affiliations.

## Supplementary Material

Supplementary Information

## Figures and Tables

**Table 1:** RaxX allele variations in the *Xanthomonas oryzae* pv. *oryzae* strains.

Strains	3	4	9	17	20	27	44	46	48	55	57
BXO1	H	S	T	R	G	P	P	A	P	R	P
IXO884						Q					
IXO1088/IXO1104/DXO331/XOOP			A								
IXO651/IXO685/IXO1221	L			W	R		S	P	T	P	N
BXOR1/MAI10/CFBP7342	L						S	P	T	P	N
BLS256	L	L					S	P	T	P	N

Various amino acid residues in different strains are compared and variations are listed with reference to BXO1 strain.
