# Supplementary material for: Population genomic insights into variation and evolution of Xanthomonas oryzae pv. oryzae
Source: Sci Rep. 2017 Jan 13;7:40694. doi: 10.1038/srep40694 (PMC5233998; doi:10.1038/srep40694)
Supplement: Supplementary Information [file srep40694-s1.pdf]

Supplementary Material

Population genomic insights into variation and evolution of *Xanthomonas oryzae* pv. oryzae

Samriti Midha, Kanika Bansal, Sanjeet Kumar, Anil Madhusoodana Girija, Deo Mishra, Kranthi Brahma, Gouri Sankar Laha, Raman Meenakshi Sundaram, Ramesh V Sonti, Prabhu B Patil*

*Corresponding author: Prabhu B. Patil

Email id: pbpatil@imtech.res.in

Supplementary Table S1: *Xanthomonas oryzae* strains: Assembly statistics, genome features, isolation details and accession numbers.

S. No.	Strain	Genome size (bp)	Coverage (x)	N50 (bp)	Contigs	Genes	tRNA	Isolation details		Pathotype	Accession No.
	***Xanthomonas oryzae* pv. oryzae strains**							Location	Year		
1	BXO1	4409965	151.1	20476	446	4294	51	West Bengal	1992	NA	JXDM00000000
2	BXO2	4239784	126.5	20109	419	4048	51	Chhattisgarh	1991	NA	JXDN00000000
3	BXO6	4274244	145.3	18946	479	4118	50	Punjab	1992	NA	JXDO00000000
4	BXO8	4316351	182.4	20644	455	4132	50	Andhra Pradesh	1993	NA	JXDP00000000
5	BXO25	4262297	325.4	23165	408	4126	52	Maharashtra	1994	NA	JXDQ00000000
6	BXO33	4259412	223.6	23280	413	4096	51	Haryana	1994	NA	JXDR00000000
7	BXO34	4287778	163.6	20348	442	4127	52	Haryana	1994	NA	JXDS00000000
8	BXO407	4371410	210.1	20047	451	4235	51	Maharashtra	1995	NA	JXDT00000000
9	BXO416	4237818	269.2	21644	411	4050	50	Haryana	1995	NA	JXDU00000000
10	BXO432	4295501	209.8	20429	455	4195	51	Maharashtra	1995	NA	JXDV00000000
11	BXO439	4240903	218.7	22710	398	4053	51	Haryana	1995	NA	JXDW00000000
12	BXO447	4276794	207.0	20041	427	4104	50	Uttarakhand	1995	NA	JXDX00000000
13	BXO454	4213466	172.1	21159	430	4037	51	Telangana	1996	NA	JXDY00000000
14	BXO471	4242472	196.4	20866	405	4082	50	Telangana	1996	NA	JXDZ00000000
15	BXO512	4261893	209.1	21113	402	4086	51	Telangana	1996	NA	JXEA00000000
16	BXO554	4289907	219.4	20876	437	4123	50	Telangana	1997	NA	JXEB00000000
17	BXO557	4251207	150.6	20582	422	4054	50	Telangana	1997	NA	JXEC00000000
18	BXO558	4258654	205.4	20739	400	4076	51	Telangana	1997	NA	JXED00000000
19	BXO559	4249121	140.1	20486	449	4088	51	Telangana	1997	NA	JXEE00000000
20	BXO571	4269282	256.1	20847	435	4120	48	Telangana	1997	NA	JXEF00000000
21	BXO582	4271769	221.9	20620	416	4105	50	Telangana	1997	NA	JXEG00000000
22	BXO589	4255070	190.5	20619	418	4091	51	Telangana	1997	NA	JXEH00000000
23	BXO590	4219024	228.8	22657	418	4046	47	Telangana	1997	NA	JXEI00000000
24	DXO-012	4262859	149.9	19970	425	4087	51	Chhattishgarh	2002	NA	JXEJ00000000

25	DXO-015	4275156	111.7	19856	485	4094	50	Tamil Nadu	2002	NA	JXEK00000000
26	DXO-27	4224373	219.1	21233	432	4074	44	West Bengal	2003	NA	JXEL00000000
27	DXO-044	4251145	145.3	20528	418	4087	51	Telangana	2003	NA	JXEM00000000
28	DXO-50	4354764	201.4	22579	414	4218	50	Andhra Pradesh	2003	NA	JXEN00000000
29	DXO-052	4237907	155.3	20970	430	4040	51	Himachal Pradesh	2004	NA	JXEO00000000
30	DXO-089	4293383	132.1	19455	450	4118	51	Gujarat	2005	NA	JXEP00000000
31	DXO-091	4290334	178.4	21886	405	4121	44	Karnataka	2005	NA	JXEQ00000000
32	DXO-116	4255751	107.7	20045	439	4096	50	Maharashtra	2006	NA	JXER00000000
33	DXO-122	4264301	194.6	21814	410	4081	51	West Bengal	2006	NA	JXES00000000
34	DXO-129	4253703	124.9	21823	413	4057	51	Punjab	2006	NA	JXET00000000
35	DXO-131	4245743	194.4	21158	432	4058	51	Bihar	2006	NA	JXEU00000000
36	DXO-133	4281787	107.1	20801	420	4091	51	Chhattishgarh	2006	NA	JXEV00000000
37	DXO-150	4237140	174.4	22619	432	4077	50	Kerala	2006	NA	JXEW00000000
38	DXO-165	4303937	154.2	21691	425	4155	51	Haryana	2008	NA	JXEX00000000
39	DXO-170	4328946	252.5	22673	408	4184	51	Punjab	2008	NA	JXEY00000000
40	DXO-174	4298137	172.6	22127	448	4097	48	Maharashtra	2008	NA	JXEZ00000000
41	DXO-181	4275923	172.0	22275	436	4134	51	Uttar Pradesh	2009	NA	JXFA00000000
42	DXO-200	4248364	129.7	20138	459	4042	51	Uttarakhand	2009	NA	JXFB00000000
43	DXO-203	4257948	312.3	23083	392	4088	51	Assam	2009	NA	JXFC00000000
44	DXO-206	4378119	122.0	22696	431	4216	51	Tripura	2009	NA	JXFD00000000
45	DXO-216	4267293	186.7	21678	412	4087	50	Haryana	2010	NA	JXFE00000000
46	DXO-226	4275008	151.6	22269	443	4128	51	Punjab	2010	NA	JXFF00000000
47	DXO-233	4382264	215.5	22537	472	4201	51	Punjab	2010	NA	JXFG00000000
48	DXO-242	4276897	168.0	22586	478	4132	49	Uttarakhand	2010	NA	JXFH00000000
49	DXO-246	4284317	147.7	20809	467	4092	50	Uttarakhand	2010	NA	JXFI00000000
50	DXO-248	4228504	124.3	22516	419	4033	47	Andhra Pradesh	2010	NA	JXFJ00000000
51	DXO-331	4319758	164.1	23827	430	4188	50	North Andaman	2012	NA	JXFK00000000
52	DXO-369	4262607	185.8	23071	405	4093	50	Andhra Pradesh	2013	NA	JXFL00000000
53	DXO-397	4262687	159.0	21870	424	4118	50	Tamil Nadu	2014	NA	JXFM00000000

54	IXO35	4337327	156.5	23206	408	4173	51	Chhattisgarh	2004	IV	JXFN00000000
55	IXO74	4314139	190.3	21881	424	4166	44	Chhattisgarh	2004	III	JXFO00000000
56	IXO89	4297350	106.3	20374	448	4096	50	Uttarakhand	2004	II	JXFP00000000
57	IXO90	4296770	150.3	21965	477	4158	52	Uttar Pradesh	2004	VIII	JXFQ00000000
58	IXO92	4310827	207.9	20763	419	4172	49	Punjab	2005	IV	JXFR00000000
59	IXO93	4272049	145.8	22676	418	4128	51	Uttar Pradesh	2005	IX	JXFS00000000
60	IXO97	4327303	236.0	23272	430	4185	51	Tamil Nadu	2005	IX	JXFT00000000
61	IXO98	4256238	247.5	22667	416	4107	52	Himanchal Pradesh	2005	IV	JXFU00000000
62	IXO99	4275556	152.1	20446	405	4107	47	Andhra Pradesh	2005	V	JXFV00000000
63	IXO134	4396360	235.3	23640	438	4289	51	Maharashtra	2005	V	JXFW00000000
64	IXO141	4346133	139.6	20314	432	4239	50	Odisha	2005	VIII	JXFX00000000
65	IXO151	4363227	321.8	22393	474	4254	51	Haryana	2005	X	JXFY00000000
66	IXO159	4282480	223.4	22702	426	4154	51	Uttar Pradesh	2005	IX	JXFZ00000000
67	IXO189	4280799	235.3	23946	410	4134	51	West Bengal	2005	III	JXGA00000000
68	IXO220	4299230	105.6	20950	450	4148	51	Punjab	2005	V	JXGB00000000
69	IXO221	4252034	113.2	20455	433	4062	48	Andhra Pradesh	2005	I	JXGC00000000
70	IXO222	4368922	187.2	20944	456	4247	51	Odisha	2005	VII	JXGD00000000
71	IXO278	4310672	280.4	20704	448	4137	50	Uttar Pradesh	2005	I	JXGE00000000
72	IXO365	4390245	177.2	19611	477	4285	51	Odisha	2005	VIII	JXGF00000000
73	IXO367	4254067	152.9	22063	420	4115	51	Uttarakhand	2006	IV	JXGG00000000
74	IXO390	4357721	176.9	22264	440	4254	47	Odisha	2005	VII	JXGH00000000
75	IXO411	4303321	205.1	22192	439	4140	51	Chhattisgarh	2006	II	JXGI00000000
76	IXO414	4241926	135.5	20529	420	4037	51	Bihar	2006	V	JXGJ00000000
77	IXO493	4381461	140.7	20059	466	4268	51	Odisha	2006	VIII	JXGK00000000
78	IXO597	4280187	169.4	21257	445	4159	51	Uttar Pradesh	2005	VII	JXGL00000000
79	IXO599	4306356	188.8	21207	433	4168	51	Uttar Pradesh	2005	VII	JXGM00000000
80	IXO603	4279185	153.5	21345	444	4151	51	Odisha	2007	IX	JXGN00000000
81	IXO608	4276528	181.5	22243	423	4128	51	Odisha	2007	IX	JXGO00000000
82	IXO620	4359881	266.1	22195	410	4177	51	Odisha	2005	VI	JXGP00000000

83	IXO621	4344066	290.7	22360	430	4187	50	Odisha	2005	VI	JXGQ00000000
84	IXO627	4332145	124.0	21365	496	4167	48	Odisha	2007	NA	JXGR00000000
85	IXO630	4407844	236.2	21137	481	4290	51	Odisha	2005	VI	JXGS00000000
86	IXO639	4275115	135.7	22616	404	4113	51	Punjab	2006	III	JXGT00000000
87	IXO644	4377298	263.9	22687	432	4253	51	Odisha	2005	VI	JXGU00000000
88	IXO645	4406524	130.7	21190	490	4307	50	Odisha	2005	X	JXGV00000000
89	IXO651	4343745	137.3	20493	457	4166	50	Punjab	2006	II	JXGW00000000
90	IXO675	4296333	220.2	20655	433	4127	51	Punjab	2007	I	JXGX00000000
91	IXO685	4371581	166.4	20066	474	4213	51	Kerala	2006	II	JXGY00000000
92	IXO704	4313810	109.7	19686	459	4157	51	Punjab	2007	X	JXGZ00000000
93	IXO725	4378616	223.9	21018	453	4232	51	Maharashtra	2007	II	JXHA00000000
94	IXO792	4369509	223.5	20845	448	4246	51	Chhattisgarh	2007	VII	JXHB00000000
95	IXO812	4325706	273.9	20436	474	4187	50	Andhra Pradesh	2007	I	JXHC00000000
96	IXO842	4349235	221.3	20359	466	4217	51	Chhattisgarh	2007	X	JXHD00000000
97	IXO884	4324742	151.0	20503	450	4133	50	Maharashtra	2008	II	JXHE00000000
98	IXO1088	4401239	157.8	20277	475	4257	50	Tripura	2009	XI	JXHF00000000
99	IXO1104	4395012	235.8	21739	475	4270	51	Tripura	2009	XI	JXHG00000000
100	IXO1221	4343762	184.4	20840	490	4227	48	Haryana	2011	NA	JXHH00000000
	***Xanthomonas oryzae* pv. oryzicola strain**										
101	BXOR1	4384839	173.8	46670	276	4101	52	Telangana	1996	NA	JXHI00000000

Supplementary Table S2: Likelihood value for the test run using two different algorithms for strict clock and relaxed clock model.

S.No.	Algorithm	Strict Clock	Relaxed Clock
1	Markov chain Monte Carlo (MCMC)		
	Harmonic Mean	-4.961868e+06	-4.959007e+06
2	Stepping Stones (SS)		
	Marginal Likelihood	-4.962580e+06	-4.958650e+06

Supplementary Table S3: Type III effector conservation status in *Xanthomonas oryzae* strains. Each lineage is represented in different colour.

		AvrBs2	HpaA	XopA	XopAA	XopAB	XopAD	XopAE	XopC	XopF	XopG	XopI	XopK	XopL	XopN	XopP	XopQ	XopR	XopT	XopU	XopV	XopW	XopX	XopY	XopZ
1	XO_X8-1A	C	C	P	C	C	P	C	C	C	A	C	C	C	C	C	C	C	P	A	C	C	C/F	C	C
2	XO_X11-5A	C	C	C	C	C	P	C	C	C	A	C	C	C	C	C	C	C	P	A	C	C	C/F	C	C
3	XOO_NAI8	C	C	C	P	C	P	C	C	C	A	C	C	C	C	P	C	C	A	C	C	C	C/F	C	C
4	XOC_MAI10	C	C	C	P	C	P	C	C	P	A	C	C	C	C	P	C	C	A	C	C	P	C/F	C	C
5	XOC_BXOR1	C	C	C	C	C	P	C	C	C	A	C	C	C	C	C	C	C	A	C	C	C	C/F	C	C
6	XOC_BLS256	C	C	C	C	C	P	C	C	C	A	C	C	C	C	C	C	C	A	C	C	C	C/F	C	C
7	XOC_CFBP7342	C	C	C	C	C	C	C	C/F	C	A	C	C	C	C	C	C	C	A	C	C	C	C/F	C	C
8	IXO597	C	C	C	C	C	P	C	C	C	A	C	C	C	C	C	C	C	P	C	C	C	C	C	C
9	IXO599	C	C	C	C	C	C	C	C	C	A	C	C	C	C	C	C	C	P	C	C	C	P	C	C
10	DXO-216	C	C	C	C	C	C	C	C	C	A	C	C	C	C	C	C	C	C/F	P	C	P	C	C	C
11	DXO-331	C	C	C	C	C	C	C	C	C	C	C	C	C	C	C	C	C	C	C	C	C	C/F	C	C
12	DXO-181	C	C	C	C	C	C	C	C	C	C	C	C	C	C	C	C	C	P	C	C	P	P	C	P
13	DXO-226	C	C	C	C	C	C	C	C	C	C	C	C	C	C	C	C	C	P	C	C	C	C	C	C
14	XOOM	C	C	C	C	C	C	C	C	C	C	C	C	C	C	C	C	C	C	C	C	C	C	C	C
15	IXO90	C	C	C	C	C	P	C	C	C	C	C	C	C	C	C	C	C	C	P	C	P	C	C	C
16	IXO159	C	C	C	C	C	C	C	C	C	C	C	C	C	C	C	C	C	C/F	C	C	C	C	C	C
17	IXO93	C	C	C	C	C	C	C	C	C	C	C	C	C	C	C	C	C	C	C	C	C	C	C	C
18	DXO-206	C	C	C	C	C	C	C	C	C	C	C	C	C	C	C	C	C	C	C	C	P	C	C	C
19	IXO97	C	C	C	C	C	C	C	C	C	A	C	C	C	C	C	C	C	C	C	C	A	C	C	C
20	IXO603	C	C	C	C	C	C	C	C	C	C	C	C	C	C	C	C	C	C	C	C	C	C	C	C
21	IXO608	C	C	C	C	C	C	C	C	C	C	C	P	C	C	C	C	C	C	C	C	C	C	C	C
22	XOOK	C	C	C	C	C	C	C	C	C	C	C	C	C	C	C	C	C	C	C/F	C	C	C	C	C
23	PXO83	C	C	C	C	C	C	C	C	C	A	C	C	C	C	C	C	C	C/F	C	C	C	C	C	C
24	PXO86	C	C	C	C	C	C	C	C	C	A	C	C	C	C	C	C	C	C/F	C	C	C	C	C	C

25	DXO-015	C	C	C	C	C	C	C	C	C	C/F	C	C	C	C	C	C	C	C/F	C	C	C	C/F	C	C
26	IXO411	C	C	C	C	P	C	C	C	C	C/F	C	C	C	C	C	C	C	C/F	C	C	C	C/F	C	C
27	DXO-246	C	C	C	C	C	C	C	C	C	C/F	C	C	C	C	C	C	C	C/F	C	C	C	C/F	C	C
28	DXO-200	C	C	P	C	C	C	C	C	C	C/F	C	C	C	C	C	C	C	C/F	C	C	C	C/F	C	C
29	DXO-242	C	C	C	C	C	C	C	C	C	C/F	C	C	C	C	C	C	C	C/F	C	C	P	C/F	C	P
30	IXO675	C	C	C	C	C	C	C	C	C	C/F	C	C	C	C	C	C	C	A	C	C	C	C/F	C	C
31	IXO278	C	C	C	C	C	C	C	C	C	C/F	C	C	C	C	C	C	C	C/F	C	C	A	C/F	C	C
32	XO35933	C	C	C	C	C	C	C	C	C	A	C	C	C	C	P	C	C	P	C	C	C	C	C	C
33	XOOP	C	C	C	C	C	C	C	C	C	P	C	C	C	C	C	C	C	A	C	C	C	C	C	C
34	IXO884	C	C	C	C	C	C	C	C	C	C/F	C	C	C	C	C	C	C	A	C	C	C	C	C	C
35	BXO8	C	C	P	C	P	C	C	P	C	C/F	C	C	C	C	C	C	C	C/F	C	C	C	C	C	C
36	IXO1221	C	C	C	C	P	C	C	C	C	C/F	C	C	C	C	C	C	C	A	C	C	C	C	C	C
37	IXO685	C	C	P	C	C	P	C	C	C	C/F	C	C	C	C	C	C	C	C/F	C	C	C	C	C	C
38	IXO651	C	C	P	C	P	C	C	C	C	C/F	C	C	C	C	C	C	C	P	C	C	A	C	C	C
39	IXO725	C	C	C	C	P	C	C	C	C	A	C	C	C	C	C	C	C	C/F	C	C	P	C	C	C
40	BXO6	C	C	C	C	C	C	C	C	C	C/F	C	C	C	C	C	C	C	A	C	C	C	C/F	C	C
41	IXO89	C	C	P	C	C	C	C	C	C	A	C	C	C	C	C	C	C	A	C	C	C	C	C	C
42	IXO1088	C	C	C	C	C	C	C	C	C	C/F	C	C	C	C	C	C	C	A	P	C	C	C	C	C
43	IXO1104	C	C	C	C	C	C	C	C	C	C/F	C	C	C	C	C	C	C	C/F	C	C	P	C	C	P
44	DXO-174	C	C	C	C	A	C	C	C	C	C/F	C	C	C	C	C	C	C	C/F	P	C	C	C	C	P
45	DXO-233	C	C	C	C	C	C	C	C	C	C/F	C	C	C	C	C	C	C	C/F	P	C	C	C	C	C
46	IXO141	C	C	C	C	C	C	C	C	C	C	C	C	P	C	C	C	C	C	C	C	C	C	C	C
47	IXO390	C	C	C	C	C	C	C	C	C	C	C	C	P	C	C	C	C	C	C	C	C	C	C	C
48	IXO621	C	C	C	C	C	C	C	C	C	C	C	C	C	C	C	C	C	C/F	C	C	A	P	C	C
49	IXO620	C	C	C	C	C	C	C	C	C	C	C	C	C	C	C	C	C	C/F	C	C	C	C	C	C
50	IXO644	C	C	C	C	C	C	C	C	C	C	C	C	C	C	C	C	C	C/F	C	C	P	C	C	C
51	IXO645	C	C	C	C	C	C	C	C	C	C	C	C	C	C	C	C	C	P	P	C	C	C	C	C
52	IXO704	C	C	C	C	C	C	C	C	C	C	C	C	C	C	C	C	C	A	P	C	P	C	C	C
53	IXO842	C	C	C	C	C	C	C	C	C	C	C	C	C	C	C	C	C	P	C	C	C	C	C	C

54	IXO222	C	C	C	C	C	C	C	C	C	C	C	C	C	C	C	C	C	C/F	P	C	C	C	C	P
55	IXO792	C	C	C	C	C	C	C	C	C	C	C	C	C	C	C	C	C	P	C	C	C	C	C	C
56	IXO630	C	C	C	C	C	C	C	C	C	C	C	C	C	C	C	C	C	A	C	C	C	C	C	C
57	IXO365	C	C	C	C	C	C	C	C	C	C	C	C	C	C	C	C	C	P	P	C	P	C	C	C
58	IXO493	C	C	C	C	C	C	C	C	C	C	C	C	C	C	C	C	C	P	P	C	C	C	C	C
59	DXO-129	C	C	C	C	C	C	C	C	C	C	C	C	C	C	C	C	C	C/F	P	C	C	C	C	C
60	DXO-165	C	C	C	C	C	C	C	C	C	C	C	C	C	C	C	C	C	C/F	C	C	C	C	C	C
61	BXO25	C	P	P	C	C	C	C	C	A	C	C	C	C	C	C	C	C	C/F	C	C	P	C	C	C
62	BXO33	C	C	C	C	C	C	C	C	C	C	C	C	C	C	C	C	C	A	C	C	C	C	C	C
63	IXO151	C	C	C	C	C	C	C	C	C	C	C	C	C	C	C	C	C	C/F	C	C	C	C	C	C
64	BXO447	C	C	C	C	C	C	C	C	C	C	C	C	C	C	C	C	C	C/F	C	C	C	C	C	C
65	BXO557	C	C	C	C	C	C	C	C	C	C	C	C	C	C	C	C	C	C/F	P	C	P	C/F	C	C
66	DXO-170	C	C	C	C	C	C	C	C	C	C	C	C	C	C	C	C	C	C/F	C	C	C	C/F	C	C
67	DXO-089	C	C	C	C	C	C	C	C	C	C	C	C	C	C	C	C	C	C/F	C	C	C	C/F	C	C
68	IXO220	C	C	C	C	C	C	C	C	C	C	C	C	C	C	C	C	C	C/F	C	C	P	C/F	C	C
69	DXO-052	C	C	C	C	C	C	C	C	C	C	C	C	C	C	C	C	C	A	C	C	C	C/F	C	P
70	BXO34	C	C	C	C	C	C	C	C	C	C	C	C	C	P	C	C	C	C/F	C	C	C	C/F	C	C
71	IXO92	C	C	C	C	C	C	C	C	C	C	C	C	C	C	C	C	C	C/F	C	C	C	C/F	C	C
72	BXO416	C	C	C	C	C	C	C	C	C	C	C	C	C	C	C	C	C	C/F	C	C	P	C/F	C	C
73	BXO439	C	C	C	C	C	C	P	C	C	C	C	C	C	C	C	C	C	C/F	P	C	C	C	C	C
74	IXO189	C	C	C	C	C	C	C	C	C	C	C	C	C	C	C	C	C	C/F	P	C	C	C/F	C	C
75	IXO98	C	C	C	C	C	C	C	C	C	C	C	C	C	C	C	C	C	C/F	C	C	C	C/F	C	P
76	IXO627	C	C	C	C	C	C	C	C	C	C	C	C	C	C	C	C	C	C/F	C	C	C	C	C	C
77	IXO639	C	C	C	C	C	C	C	C	C	C	C	C	C	C	C	C	C	C/F	C	C	C	C/F	C	C
78	IXO134	C	C	C	C	C	C	C	C	C	A	C	C	C	C	C	C	C	C/F	C	C	A	C	C	C
79	IXO99	C	C	C	C	C	P	C	C	C	C	C	C	C	C	C	C	C	C/F	C	C	C	C	C	C
80	BXO512	C	C	C	C	C	C	C	C	C	C	C	C	C	C	C	C	C	C/F	P	C	C	C	C	C
81	DXO-044	C	C	C	C	C	C	C	C	C	C	C	C	C	C	C	C	C	C/F	C	C	C	C	C	C
82	DXO-027	C	C	C	C	C	C	C	C	C	C	C	C	C	C	C	C	C	A	P	C	C	C	C	C

83	BXO407	C	C	P	C	C	C	C	C	C	C	C	C	C	C	C	C	C	C/F	C	C	C	C	C	C
84	BXO432	C	C	C	C	C	C	C	C	C	C	C	C	C	C	C	C	C	C/F	C	C	C	C	C	P
85	DXO-397	C	C	C	C	C	C	C	C	C	C	C	C	C	C	C	C	C	C/F	P	C	C	C	C	C
86	BXO589	C	C	C	C	C	C	C	C	C	C	C	C	C	C	C	C	C	C/F	C	C	C	C	C	C
87	IXO221	C	C	C	C	C	C	C	C	C	C	C	C	C	C	C	C	C	C/F	C	C	C	C	C	C
88	DXO-131	C	C	C	C	C	C	C	C	C	C	C	C	C	C	C	C	C	C/F	P	C	C	C	C	C
89	IXO812	C	C	C	C	C	C	C	C	C	C	C	C	C	C	C	C	C	C	C	C	C	C	C	C
90	BXO554	C	C	C	C	C	C	C	C	C	C	C	C	C	C	C	C	C	C/F	C	C	C	C	C	C
91	BXO590	C	C	C	C	C	C	C	C	C	C	C	C	C	C	C	C	C	C/F	C	C	C	C	C	P
92	DXO-116	C	C	C	C	C	C	C	C	C	C	C	C	C	C	C	C	C	C/F	P	C	C	C	C	C
93	DXO-050	C	C	C	C	C	C	C	C	C	C	C	C	C	C	C	C	C	C/F	P	C	P	C	C	C
94	BXO471	C	C	C	C	C	C	C	C	C	C	C	C	C	C	C	C	C	C/F	C	C	C	C	C	C
95	DXO-091	C	C	C	C	C	C	C	C	C	C	C	C	C	C	C	C	C	C/F	C	C	C	C	P	C
96	BXO1	C	C	C	C	C	C	C	C	C	C	C	C	C	C	C	C	C	C/F	C	C	C	C	C	C
97	IXO367	C	C	C	C	C	C	C	C	C	C	C	C	C	C	C	C	C	P	C	C	C	C	C	C
98	DXO-150	C	C	C	C	C	C	P	C	C	C	C	C	C	C	C	C	C	C/F	C	C	C	C	C	C
99	DXO-203	C	C	C	C	C	P	C	C	C	C	C	C	C	C	C	C	C	A	C	C	C	C	C	C
100	DXO-248	C	C	C	C	C	C	C	C	C	C	C	C	C	C	C	C	C	C/F	C	C	C	C	C	C
101	DXO-369	C	C	C	C	C	C	C	C	C	C	C	C	C	C	C	C	C	C/F	P	C	C	C	C	C
102	BXO571	C	C	C	C	C	C	C	C	C	C	C	C	C	C	C	C	C	C/F	P	C	C	C	C	C
103	DXO-133	C	C	C	C	C	C	C	C	C	C	C	C	C	C	C	C	C	C/F	C	C	C	C	C	C
104	BXO582	C	C	C	C	C	C	C	C	C	C	C	C	C	C	C	C	C	C/F	C	C	C	C	C	C
105	IXO414	C	C	C	C	C	C	C	C	C	C	C	C	C	C	C	C	C	C/F	C	C	C	C	C	C
106	BXO2	C	C	C	C	C	C	C	C	C	C	C	C	C	C	C	C	C	C	C	C	C	C	C	C
107	DXO-122	C	C	C	C	C	C	C	C	C	C	C	C	C	C	C	C	C	C/F	C	C	C	C	C	C
108	DXO-012	C	C	C	C	C	C	C	C	C	C	C	C	C	C	C	C	C	C/F	P	C	C	C	C	C
109	BXO558	C	C	C	C	C	C	C	C	C	C	C	C	C	C	C	C	C	C/F	P	C	C	C	C	C
110	BXO454	C	C	C	C	C	C	C	C	C	C	C	C	C	C	C	C	C	A	C	C	C	C	C	C
111	BXO559	C	C	C	C	C	C	C	C	C	C	C	A	P	C	C	C	C	A	C	C	C	C	C	P

112	IXO35	C	C	C	C	C	C	C	C	C	C	C	C	C	C	C	C	C	C/F	C	C	C	C	C	C
113	IXO74	C	C	C	C	C	C	C	C	C	C	C	C	C	C	C	C	C	C/F	C	C	C	C	C	C

C Complete

P Partial

A Absent

C/F Complete with frame shift

Supplementary Table S4: CRISPR locus with their number of spacers in *Xanthomonas oryzae* pv. oryzae strains. Each lineage is represented in different colour. Repeats (1-3) represent the same CRISPR locus assembled as a part of different contigs.

Repeat Sequence: GTTTCAATCCACGCGCCCGTGAGGACGCGAC

S. No.	Strain	Repeats-1	Repeats-2	Repeats-3	Total number of spacers
1	IXO597	23			22
2	IXO599	23			22
3	DXO-216	23			22
4	DXO-331	77			76
5	DXO-181	77			76
6	DXO-226	79			78
7	XOOM	49			48
8	IXO90	38			37
9	IXO159	45			44
10	IXO93	45			44
11	DXO-206	43			42
12	IXO97	42			41
13	IXO603	42			41
14	IXO608	42			41
15	XOOK	60			59
16	PXO83	86			85
17	PXO86	88			87
18	DXO-015	29			28
19	IXO411	29			28
20	DXO-246	29			28
21	DXO-200	29			28
22	DXO-242	29			28
23	IXO675	29			28
24	IXO278	29			28
25	XO35933	0			0
26	XOOP	76			75
27	IXO884	70			69
28	BXO8	0			0
29	IXO1221	34			33
30	IXO685	24			23
31	IXO651	34			33
32	IXO725	33			32
33	BXO6	66			65
34	IXO89	54	19		72
35	IXO1088	91	19		109
36	IXO1104	91	19		109
37	DXO-174	70	19		88
38	DXO-233	85			84
39	IXO141	77			76

40	IXO390	77			76
41	IXO621	59			58
42	IXO620	59			58
43	IXO644	59			58
44	IXO645	59			58
45	IXO704	65			64
46	IXO842	65			64
47	IXO222	65			64
48	IXO792	65			64
49	IXO630	65			64
50	IXO365	65			64
51	IXO493	65			64
52	DXO-129	53	6		58
53	DXO-165	48	6		53
54	BXO25	53			52
55	BXO33	51			50
56	IXO151	49			48
57	BXO447	89			88
58	BXO557	83	9		91
59	DXO-170	96			95
60	DXO-089	94			93
61	IXO220	94			93
62	DXO-052	97			96
63	BXO34	92			91
64	IXO92	92			91
65	BXO416	93			92
66	BXO439	93			92
67	IXO189	87			86
68	IXO98	87			86
69	IXO627	87			86
70	IXO639	87			86
71	IXO134	88			87
72	IXO99	92			91
73	BXO512	93			92
74	DXO-044	93			92
75	DXO-027	55			54
76	BXO407	92			91
77	BXO432	92			91
78	DXO-397	91			90
79	BXO589	88			87
80	IXO221	86			85
81	DXO-131	89			88
82	IXO812	90			89
83	BXO554	88			87

84	BXO590	88			87
85	DXO-116	62	5	30	96
86	DXO-050	90			89
87	BXO471	90			89
88	DXO-091	91			90
89	BXO1	91			90
90	IXO367	90			89
91	DXO-150	98			97
92	DXO-203	92			91
93	DXO-248	91			90
94	DXO-369	87			86
95	BXO571	90			89
96	DXO-133	90			89
97	BXO582	90			89
98	IXO414	89			88
99	BXO2	91			90
100	DXO-122	87			86
101	DXO-012	90			89
102	BXO558	58	31		88
103	BXO454	89			88
104	BXO559	90			89
105	IXO35	90			89
106	IXO74	90			89

Supplementary Table S5: Assembly statistics and BLAST results of Plasmids detected using plasmidSpades

			Assembly Statistics							
			Plasmid Assembly			Genome Assembly		BLAST Results		
S. No.	Strain	Query	Coverage (x)	Size (bp)	Contig No.	Coverage (x)	Size (bp)	Identity (%)	Coverage (%)	E-Value
		**Plasmid type 1: *Xanthomonas albilineans* str. GPE PC73, plasmid plasmIII (Accession number: FP340277.1)**								
1	**BXO1**	NODE_1_length_25761_cov_100.085_component_1	100.085	25761	29	213.2337	25623	95	97	0
2	**IXO704**	NODE_1_length_25761_cov_85.7783_component_1	85.7783	25761	99	183.9723	25634	95	97	0
3	**IXO842**	NODE_1_length_25761_cov_259.643_component_1	259.643	25761	79	573.6597	25634	95	97	
4	**IXO35**	NODE_2_length_25779_cov_140.858_component_2	140.858	25779	8	299.5422	25633	95	97	0
		**Plasmid type 2: *Xanthomonas campestris* pv. campestris B1459, plasmid I (Accession number LN811400.1)**								
1	**DXO-050**	NODE_1_length_40132_cov_131.765_component_0	131.765	40132	37	296.06	35705	94	77	0
2	**DXO-091**	NODE_1_length_41951_cov_117.697_component_1	117.697	41951	6	258.1336	41844	94	74	0
3	**DXO-133**	NODE_1_length_41350_cov_57.6095_component_1	57.6095	41350	189	127.6487	35968	94	75	0
6	**IXO74**	NODE_1_length_41350_cov_133.073_component_1	133.073	41350	237	296.6875	36988	94	75	0
7	**IXO97**	NODE_2_length_35857_cov_161.625_component_0	161.625	35857	13	360.3137	36257	93	83	0

5	**IXO35**	NODE_1_length_41350_cov_112.5_component_1	112.5	41350	73	252.111	36727	94	75	0
4	**DXO-206**	NODE_3_length_21529_cov_110.672_component_2	110.672	21529	149	237.7627	20784	88	75	0
		NODE_4_length_14165_cov_91.8176_component_2	91.8176	14165	1	188.9546	13207	94	91	0
		**Plasmid type 3: *Xanthomonas citri* subsp. citri str. 306, plasmid pXAC64 (Accession number: AE008925.1)**								
1	**IXO134**	NODE_1_length_24860_cov_248.574_component_0	248.574	24860	29	476.8709	30227	96	80	0
		**Plasmid type 4: *Burkholderia vietnamiensis* G4, plasmid pBVIE04 (Accession number: CP000620.1)**								
1	**DXO-206**	NODE_1_length_44493_cov_69.0478_component_1	69.0478	44493	67	154.6604	25007	87	79	0

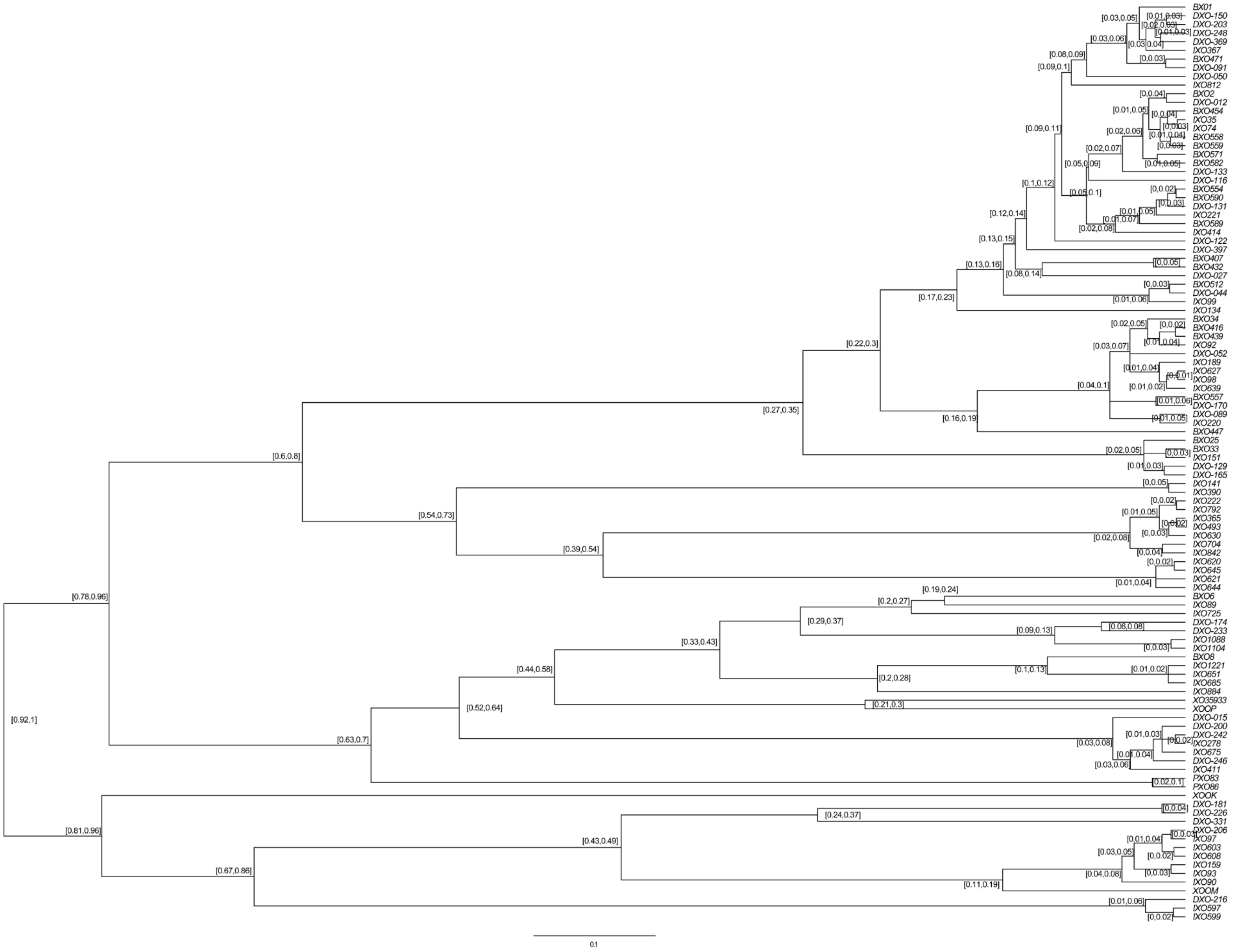
BX01
DXO-150
DXO-203
DXO-248
DXO-369
IXO367
BXO471
DXO-091
DXO-050
IXO812
BXO2
DXO-012
BXO454
IXO35
IXO74
BXO558
BXO559
BXO571
BXO582
DXO-133
DXO-116
BXO554
BXO590
DXO-131
IXO221
BXO589
IXO414
DXO-122
DXO-397
BXO407
BXO432
DXO-027
BXO512
DXO-044
IXO99
IXO134
BXO34
BXO416
BXO439
IXO92
DXO-052
IXO189
IXO627
IXO98
IXO639
BXO557
DXO-170
DXO-089
IXO220
BXO447
BXO25
BXO33
IXO151
DXO-129
DXO-165
IXO141
IXO390
IXO222
IXO792
IXO365
IXO493
IXO630
IXO704
IXO842
IXO620
IXO645
IXO621
IXO644
BXO6
IXO89
IXO725
DXO-174
DXO-233
IXO1088
IXO1104
BXO8
IXO1221
IXO651
IXO685
IXO884
XO35933
XOOP
DXO-015
DXO-200
DXO-242
IXO278
IXO675
DXO-246
IXO411
PXO83
PXO86
XOOK
DXO-181
DXO-226
DXO-331
DXO-206
IXO97
IXO603
IXO608
IXO159
IXO93
IXO90
XOOM
DXO-216
IXO597
IXO599
0.1

Supplementary Figure S1: Phylogenomic tree of 100 Indian *Xoo* strains and six *Xoo* strains from other parts of Asia along with the estimated scale of divergence. Core genome alignment of the isolates was analysed using Mr.Bayes v3.2 with GTR substitution model with gamma-distributed rate variation across sites and a proportion of invariable sites for 1M iterations (ngen=1000000) and two parallel runs (nrun=2). Molecular dating was done with clock rate (0.02, 0.004) per million year. The scale bar (0.1) indicates the number of nucleotide substitutions per site. The values indicated on the node are 95% HPD (highest-probability density) intervals for divergence time in million years.

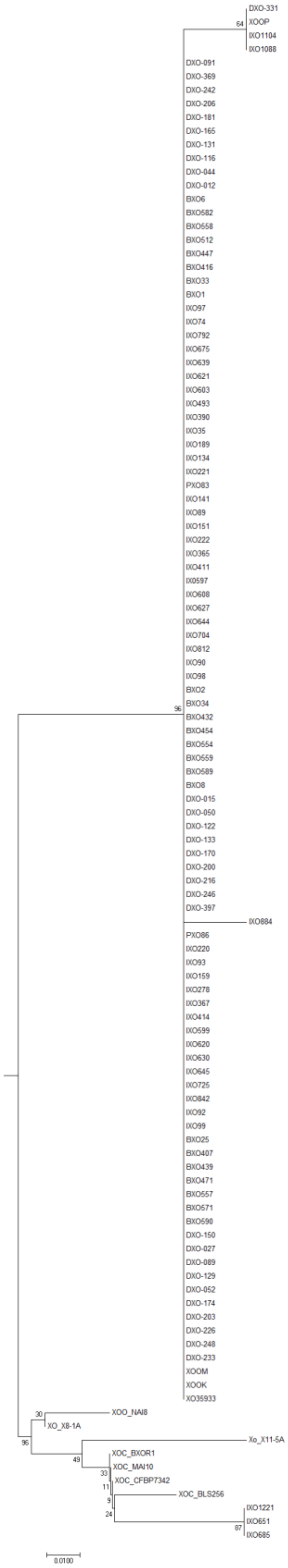

Supplementary Figure S2. Phylogenomics tree of RaxX of *Xanthomonas oryzae* strains. Protein sequences were aligned and Neighbour Joining tree of RaxX was constructed using Mega 6.0 with 500 bootstrap replicates. The scale bar (0.01) indicates the number of nucleotide substitutions per site.

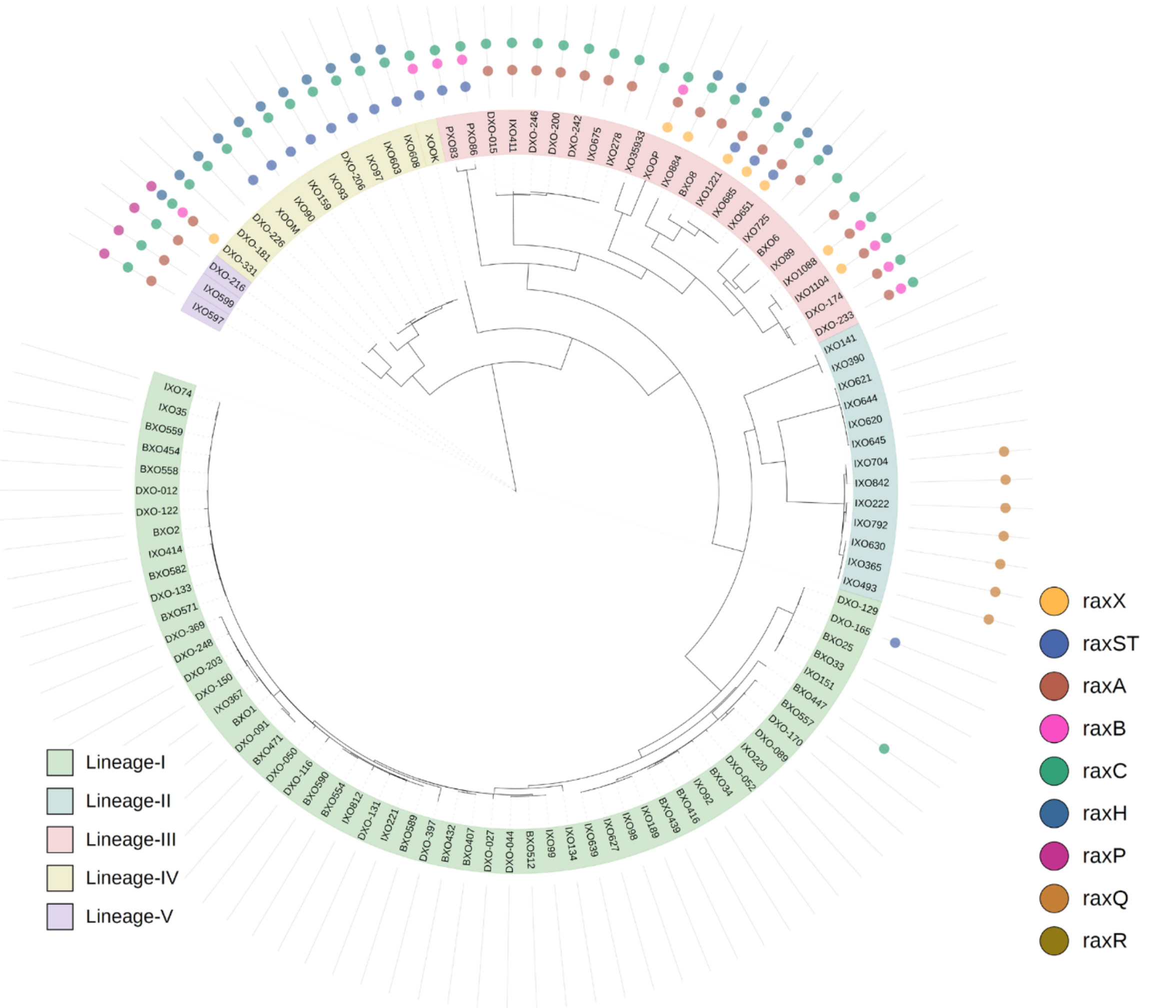

Supplementary Figure S3: Variation in *rax* genes marked on ClonalFrameML tree. Genomes of 100 Indian *Xoo* isolates and six *Xoo* strains from other parts of Asia were aligned and core genome was analysed using ClonalFrameML to obtain a tree considering recombination. Different lineages inferred are coloured differently. On the tree in circular way, information on nine *rax* genes is marked. Moving outward in the circles, the strains are marked with having non-synonymous changes in *raxX* (1), *raxST* (2), *raxA* (3), *raxB* (4), *raxC* (5), *raxH* (6), *raxP* (7), *raxQ* (8), *raxR* (9).

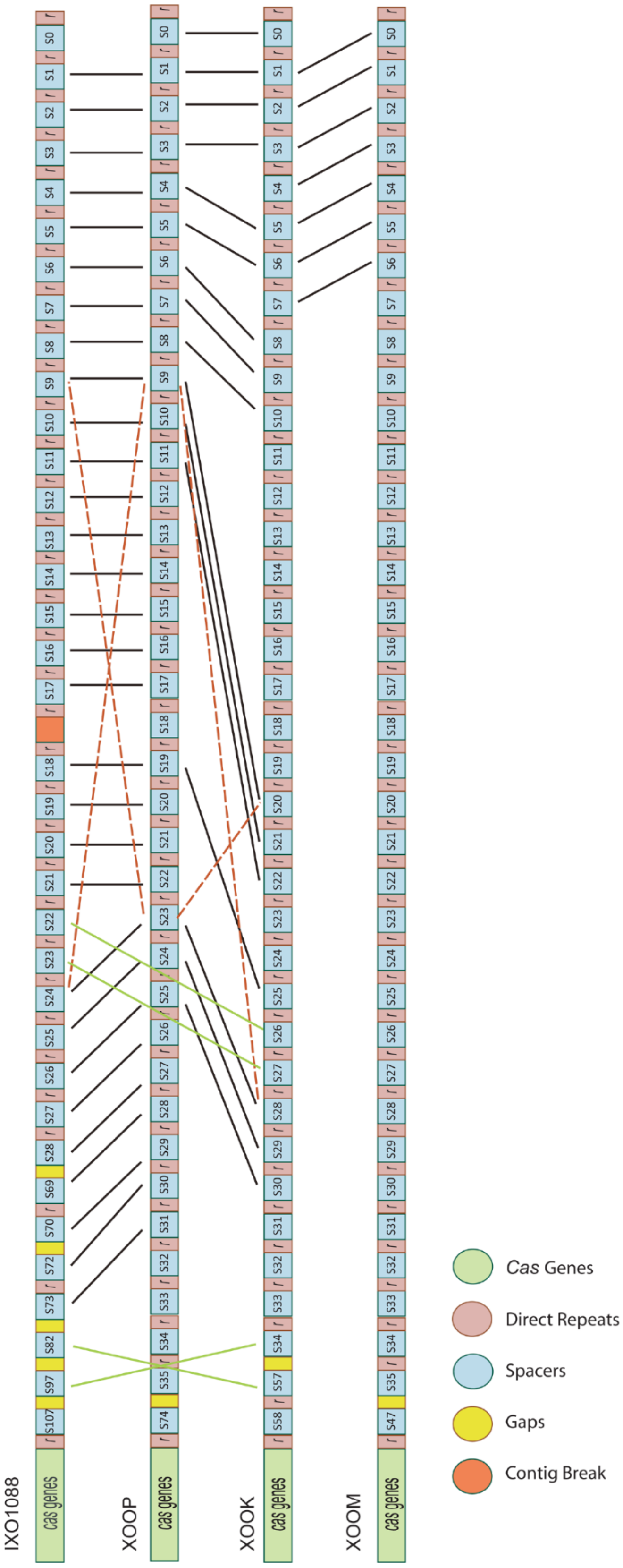
IXO1088
XOOP
XOOK
XOOM
cas genes
Cas Genes
Direct Repeats
Spacers
Gaps
Contig Break

Supplementary Figure S4: Comparison of CRISPR cassettes of *Xanthomonas oryzae* pv. *oryzae* strain IXO1088, PXO99A (XOOP), MAFF 311018 (XOOM) and KACC 10331 (XOOK). Spacers are numbered from top (S0) to bottom and presence of youngest repeat is shown towards the *cas* genes. Yellow coloured boxes show the gap, where the presence of spacers and repeats is not shown in this figure, but they are present in the genomes. Orange colour box in IXO1088, shows the break between two contigs, as the CRISPR locus did not assemble in one contig in the genome. Thick black lines link the identical spacers in two genomes shown adjacent to each other, while thick green lines link the additional similar spacers shared by IXO1088 and XOOK. Red dotted lines link the spacers which are present in multiple copies in the genome. Coordinates used: IXO1088 [Contig 248 (1874-14147) + Contig 292 (1499-1)], XOOP [4,533,334-4,546,146], XOOK [900,496-888,612] and XOOM [869,005-857,969].
